# A clinical application of fair value estimate (FVE) for R50%: Separating overlapping 50% isodose volumes in single isocenter multiple targets SRS

**DOI:** 10.1002/acm2.14061

**Published:** 2023-06-07

**Authors:** Dharmin D. Desai, Ivan L. Cordrey

**Affiliations:** ^1^ Varian Advanced Oncology Solutions Palto Alto California USA; ^2^ Regional Cancer Center Cumberland Medical Center Crossville Tennessee USA

**Keywords:** cranial SRS, cranial SRT, Fair Value Estimate, FVE, IDC50% overlap, R50%, SIMT

## Abstract

In the treatment of single isocenter multiple targets (SIMT) stereotactic cranial cases with linac‐based, multi‐leaf collimated delivery, one encounters cases when the 50% isodose clouds (IDC50%s) of planning target volumes (PTVs) in close proximity overlap and cannot easily be separated. In such cases, it is difficult to assign an IDC50% to each individual PTV, which is necessary to allow evaluation of individual PTV intermediate dose spill for comparison to established intermediate dose spill metrics for plan quality assessment. The Fair Value Estimate (FVE) for R50% (R50%_FVE_) is a method to unambiguously apportion the overlapping volume of IDC50% to allow calculation of the intermediate dose spill metric R50% (defined as volume of IDC50% / volume of PTV). Full application of R50%_FVE_ requires knowing the surface area of the PTVs. Since surface area information is not always available, we develop a spherical PTV approximation to R50%_FVE‐sphere_ and compare this to R50%_FVE_. Then we apply the R50%_FVE‐sphere_ to clinical data from the University of Alabama, Birmingham (UAB) that catalogs 68 PTVs from various SIMT plans with overlapping IDC50%. The UAB dataset reports intermediate dose spill as Falloff Index. While Falloff Index looks mathematically equivalent to R50%, the Falloff Index attributes the “entire overlapping IDC50% of PTVs in close proximity” to each individual PTV in the cluster. R50%_FVE‐sphere_ provides a value that is conceptually correct and numerically smaller relative to the Falloff Index data reported by UAB in all cases. This reprocessing of the UAB data places many of the PTVs with very high intermediate dose spill within recently proposed R50% guidelines.

## 1 INTRODUCTION

The treatment of single isocenter multiple targets (SIMT) stereotactic cranial cases with linac‐based MLC‐collimated delivery is gaining wider implementation in large academic centers and community‐based radiation therapy centers. An important goal in such treatment is ensuring that the intermediate dose spill outside the planning target volumes (PTVs) is minimized. One important metric for intermediate dose spill is R50%, defined as the ratio of the volume of the 50% isodose cloud (V_IDC50%_) to the volume of the PTV (V_PTV_).

(1) $$R50\%\;=\;\frac{V_{\mathrm{IDC}50\%}}{V_{\mathrm{PTV}}}$$

Minimizing normal brain tissue dose is also an important optimization and planning objective in cranial stereotactic treatment. Normal brain is an organ at risk (OAR) always directly adjacent to PTV surfaces and subject to the higher doses being delivered to these PTVs, and radiation necrosis of normal brain tissue is one of the more relevant adverse effects after stereotactic radiosurgery (SRS) and stereotactic radiotherapy (SRT).^1^ Brain radionecrosis has been correlated with the volume of brain that receives a dose of 12 Gy (V12Gy) for single fraction SRS and V18Gy for multi‐fraction SRT.^2^, ^3^

Intermediate dose spill is often tracked and reported in SRS/SRT studies by various metrics, such as R50% used in this study. The computation of these intermediate dose spill metrics often utilizes the volume that receives at least 50% of the prescription dose (Rx), referred to above as V_IDC50%_. Thus, V_IDC50%_ and intermediate dose spill metrics derived from it, including R50%, are reasonable surrogates for normal brain radionecrosis.

Methods for minimizing R50% and appropriate optimization goals for R50% values have been systematically developed.^4^ Evaluating the results of these R50% minimization methods depends on being able to determine the R50% of each PTV. This requires a determination of the IDC50% for each individual PTV. Yet inevitably in clinical applications, one encounters presentations of PTVs for which the PTVs are too close together to resolve the individual IDC50%. In other words, the IDC50% of closely spaced PTVs overlap or merge.

**FIGURE 1:**
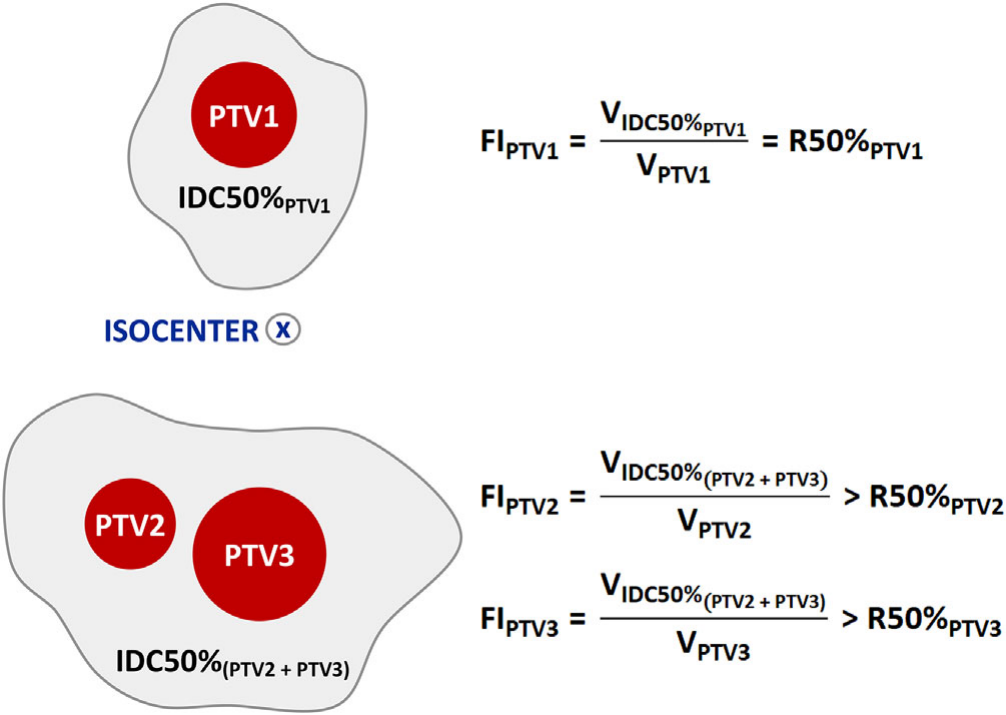
A simple illustration of a 3‐PTV SIMT case comprised of a single PTV and a 2‐PTV cluster. The Falloff Index and R50% for PTV1 are mathematically equivalent as shown in the equation for FI_PTV1_. PTV2 and PTV3 are contained in the 2‐PTV cluster and have overlapping IDC50%; therefore, IDC50%_PTV2_ and IDC50%_PTV3_ cannot be unambiguously separated. As a result, the equations for both FI_PTV2_ and FI_PTV3_ use IDC50%_(PTV2 + PTV3)_, which results in calculated Falloff Index values larger than anticipated.

As stated by Popple et al., “When targets are sufficiently close together, the 50% and 100% merge, resulting in a large value for the gradient and conformity index. For HyperArc, 10.8% of the targets had bridging at the 50% level….**^5^**” The “large values” result from assigning the entire IDC50% for the assembly of closely spaced PTVs to each individual PTV without attempting to apportion the IDC50% volume among the PTVs. This is shown in the illustration of Figure 1, which is why the metric used by Popple et al. was Falloff Index (FI), not R50%. While the Falloff Index is identical to R50% when the IDC50% associated with each individual PTV is unambiguously distinguishable, Falloff Index is different from R50% in the 10.8% of cases with overlapping IDC50% (Figure 1).

Desai and Cordrey detailed a systematic accounting system to apportion the IDC50% for those clinical cases when the IDC50% of multiple PTVs overlap, which is called the Fair Value Estimate (FVE).**^6^** FVE allows one to unambiguously assign an R50% value to each PTV in a clinical plan where the IDC50% of multiple PTVs overlaps. In so doing, FVE allows comparison with universal R50% standards published by Desai et al. for linac‐based multi‐leaf collimated stereotactic radiosurgery (SRS) and stereotactic radiation therapy (SRT).**^7^** Yet, to apply full FVE, one must know the individual PTV surface areas.^6^ Since no commercially available radiation treatment planning system (RTPS) currently reports the surface area of a segmented structure contoured in the RTPS, FVE is challenging to apply without a user‐created surface area script. This difficulty can be overcome utilizing an equivalent spherical PTV approximation as was suggested in Desai and Cordrey.^6^

This brief technical note will describe the equivalent spherical PTV approximation of FVE and apply “spherical PTV approximation FVE” to the clinical cases in the UAB dataset that used HyperArc for which the IDC50% of multiple PTVs overlapped to convert the Falloff Index into R50%.

## 2 METHODS

### 2.1 Spherical PTV approximation FVE

The full application of the FVE for R50% to a cluster of PTVs with overlapping IDC50% was described by Desai and Cordrey and requires the surface area of the individual PTVs in the cluster.^6^ The equation to calculate the R50%_FVE_ is given below:

(2) $$\begin{array}{ccc} & & {R50\%}_{\mathrm{FVE}}^{\mathrm{PT}V_{i}}={R50\%}_{\mathrm{Analytic}}^{\mathrm{PT}V_{i}}\\ & & +\frac{\frac{SA_{\mathrm{PT}V_{i}}}{\sum_{n=1}^{N}SA_{\mathrm{PT}V_{n}}}\left[V_{\mathrm{IDC}50\%}^{\mathrm{Total}}-\sum_{n=1}^{N}\left(V_{\mathrm{PT}V_{n}}{\times \;R50\%}_{\mathrm{Analytic}}^{\mathrm{PT}V_{n}}\right)\right]}{V_{\mathrm{PT}V_{i}}} \end{array}$$

where$\;SA_{\mathrm{PT}V_{i}}$ = the surface area of PTV_i_ $,\;V_{\mathrm{PT}V_{i}}$ = the volume of PTV_i_, and $V_{\mathrm{IDC}50\%}^{\mathrm{Total}}$ = the total volume of IDC50% surrounding the cluster of PTVs with overlapping IDC50%.

R50%_Analytic_ represents an “as low as reasonably achievable” value for R50% as described by Desai et al.^8^ and is given as:

(3) $$\begin{array}{c} {R50\%}_{\mathrm{Analytic}}^{\mathrm{PT}V_{i}}=1+\frac{SA_{\mathrm{PT}V_{i}}}{V_{\mathrm{PT}V_{i}}}\Delta r\left[1+\left(\frac{\Delta r}{r_{\mathrm{PTV}}}\right)+\frac{1}{3}{\left(\frac{\Delta r}{r_{\mathrm{PTV}}}\right)}^{2}\right] \end{array}$$

with

(4) $$r_{\mathrm{PTV}}\;=\;{\left(\frac{3V_{\mathrm{PTV}}}{4\pi }\right)}^{1/3}$$

(5) $$\Delta r=\;0{.2844\;\times \;V}_{\mathrm{PTV}}^{0.1973}$$

No commercially available RTPS reports the surface area of a segmented structure at this time; thus, one cannot easily apply FVE unless one has an alternative method of extracting the PTV surface area (such as a surface area script created and validated by the end user as was done by Desai et al.^8^). This limitation can be overcome by using a spherical PTV approximation to FVE, in which one assumes the PTV surface area is the surface area of a spherical PTV of the same volume as the PTV in question. Thus,

(6) $$SA_{\mathrm{PTV}-\mathrm{sphere}}\;=\;4\pi \;{\left(r_{\mathrm{PTV}}\right)}^{2}$$

Applying this approximation to Equations (2) and (3) with a little algebra, we have, respectively:

(7) $$\begin{array}{ccc} & & {R50\%}_{\mathrm{FVE}-\mathrm{sphere}}^{\mathrm{PT}V_{i}}{\;=\;R50\%}_{\mathrm{Analytic}-\mathrm{sphere}}^{\mathrm{PT}V_{i}}\\ & & +\frac{\frac{{\left(r_{\mathrm{PT}V_{i}}\right)}^{2}}{\sum_{n=1}^{N}{\left(r_{\mathrm{PT}V_{n}}\right)}^{2}}\left[V_{\mathrm{IDC}50\%}^{\mathrm{Total}}-\sum_{n=1}^{N}\left(V_{\mathrm{PT}V_{n}}{\;\times \;R50\%}_{\mathrm{Analytic}-\mathrm{sphere}}^{\mathrm{PT}V_{n}}\right)\right]}{V_{\mathrm{PT}V_{i}}} \end{array}$$

and

(8) $${R50\%}_{\mathrm{Analytic}-\mathrm{sphere}}^{\mathrm{PT}V_{i}}\;=\;1\;+\;\frac{3}{r_{\mathrm{PTV}}}\Delta r\;\left[1\;+\;\left(\frac{\Delta r}{r_{\mathrm{PTV}}}\right)+\;\frac{1}{3}{\left(\frac{\Delta r}{r_{\mathrm{PTV}}}\right)}^{2}\right]$$

with r_PTV_ and Δr given by Equations (4) and (5) above.

This spherical PTV approximation FVE (R50%_FVE‐sphere_) is now entirely in terms of the PTV volumes and the PTV surface area has been explicitly removed from the equations. To test this approximation, we apply Equation (8) to the original phantom cases studied by Desai and Cordrey.^6^ We calculate the R50%_FVE‐sphere_ for their 12 PTVs to provide a direct comparison between the R50%_FVE_ (full R50%_FVE_) and R50%_FVE‐sphere_ (spherical PTV approximation for R50%_FVE_).

### 2.2 Application of spherical PTV approximation R50%_FVE‐sphere_ to clinical data

The large clinical dataset of plans from UAB (available upon request from Popple et al.) contains both manual plans and HyperArc plans.^5^ We focus on the HyperArc plans and extract the PTVs with overlapping IDC50%. In total, 68 PTVs with merged IDC50% were extracted. There are twenty‐two, 2‐PTV clusters; four, 3‐PTV clusters; and three, 4‐PTV clusters. Details of the extracted data are provided in Appendix A.

The full application of the FVE method requires the PTV surface area; however, surface area information is not available in the UAB dataset. To address this issue, we use the R50%_FVE‐sphere_ developed above [Equation (7)].

We apply R50%_FVE‐sphere_ to each PTV cluster in the UAB dataset that had overlapping IDC50% and supply a table of reinterpreted values for the intermediate dose spill that can be cross‐referenced back to the original UAB dataset table.

## 3 RESULTS

### 3.1 Spherical PTV approximation FVE

Table 1 shows the results of R50%_FVE‐sphere_ applied to the 12 PTVs in the work of Desai and Cordrey^6^; their entire table of values is reprinted here to facilitate a direct comparison of the full R50%_FVE_ to the R50%_FVE‐sphere_ of this work. As can be seen, the differences between R50%_FVE_ and R50%_FVE‐sphere_ are not dramatic for this set of PTVs. Thus, the direct comparison of R50%_FVE_ and R50%_FVE‐sphere_ for this set of known surface area non‐spherical targets allows an assessment of the variance of R50%_FVE‐sphere_ from the full R50%_FVE_ of Desai and Cordrey.^6^

**TABLE 1:** Applying the R50%_FVE‐sphere_ accounting system.

Plan ID	Total V_IDC50%_ (cm^3^)	Remaining V_IDC50%_ (cm^3^)	PTV #	PTV Vol (cm^3^)	PTV r (cm)	Spherical PTV SA (cm^2^)	R50%_FVE‐sphere_	R50%_FVE_	Diff
2PTVs‐1Iso	29.34	16.24							
			1	1.02	0.62	4.90	7.80	8.00	−0.20
			2	3.76	0.96	11.69	5.69	5.63	0.06
2PTVsLrgSml	40.40	21.70							
			1	0.47	0.48	2.92	10.02	10.84	−0.82
			2	6.91	1.18	17.54	5.16	5.11	0.05
3PTVs‐1Iso	57.34	39.18							
			1	1.02	0.62	4.90	11.06	11.37	−0.31
			2	3.76	0.96	11.69	7.80	7.72	0.08
			3	1.75	0.75	7.02	9.55	9.55	0.00
9PTVs‐1Iso	67.49	19.70							
			2	0.72	0.56	3.88	5.81	5.60	0.21
			3	20.12	1.69	35.77	3.07	3.08	−0.01
			5	0.20	0.36	1.65	7.82	7.48	0.34
9PTVs‐1Iso	6.75	2.78							
			7	0.34	0.43	2.36	6.44	6.43	0.01
			8	0.87	0.59	4.41	5.24	5.25	−0.01

### 3.2 Application of spherical PTV approximation R50%_FVE‐sphere_ to clinical data

The full list of results for the UAB dataset HyperArc plans with overlapping IDC50% is given in Table A.1. The Falloff Index is reported exactly as provided in the UAB HyperArc dataset.^5^ The reported R50%_FVE‐sphere_ was calculated according to the Spherical PTV Approximation R50%_FVE_ of Equation (7). “Plan Index” and “Target Index” are identifiers from the original UAB dataset and allow direct correlation back to the UAB dataset.

In all cases, the R50%_FVE‐sphere_ is numerically smaller than the UAB reported Falloff Index (as it should be when the shared IDC50% volume is subdivided). The largest differences between Falloff Index and R50%_FVE‐sphere_ were seen for PTV clusters that included more PTVs. Small PTVs typically saw much larger numerical changes between Falloff Index and R50%_FVE‐sphere_ than larger PTVs. Indeed, the second 4‐PTV cluster listed for Plan Index 17 in Table A1 demonstrates this well; in this 4‐PTV cluster, the large 6 cm^3^ PTV saw very little change between Falloff Index and R50%_FVE‐sphere_ (a factor of 1.14), but the very small 0.015 cm^3^ PTV saw a dramatic change (a factor of 93).

**FIGURE 2:**
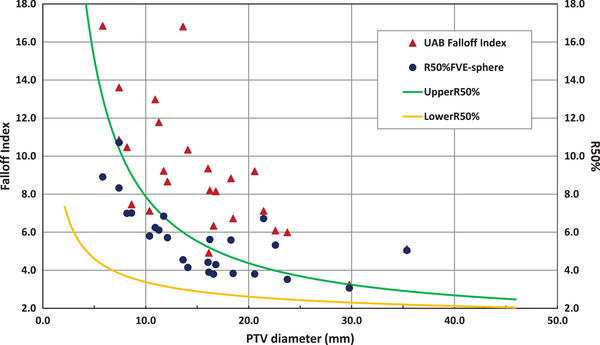
Falloff Index and R50%_FVE_ versus PTV diameter graph comparing the UAB Falloff Index values to the calculated R50%_FVE‐sphere_ values. In all cases, R50%_FVE‐sphere_ is numerically less than the UAB Falloff Index. For some extremely small volume PTVs, the difference can be dramatic; therefore, only PTVs with an initial Falloff Index < 18 are included in this graph (25 of the total 68 PTVs) to allow direct one‐to‐one comparison Falloff Index and R50%_FVE‐sphere_ for each PTV displayed. Note that for data points at PTV Diameter 35.4 mm and 29.8 mm have Falloff Index values that are nearly identical to the R50%_FVE‐sphere_ so the blue circle eclipses the red triangle making it hard to see the red triangle of the Falloff Index. The numerical values for the entire data set of 68 PTVs are listed in Table A.1. UpperR50% and LowerR50% curves are proposed intermediate dose spill guidelines (R50%)**^8^** and are displayed to provide some relative context to the changes in the values of Falloff Index and R50%_FVE‐sphere_.

Figure 2 displays a subset of the Table A.1 data for PTVs that have Falloff Index < 18 (25 of the 68 total PTVs extracted), along with the corresponding R50%_FVE‐sphere_. For reference, Figure 2 also includes the UpperR50% and LowerR50% guidelines as curves.**^7^** UpperR50% and LowerR50% are intermediate dose spill metric quality assessment guidelines proposed by Desai et al. According to Desai et al., all SRS plans should ideally have R50% values that fall within the range between UpperR50% and LowerR50%. The graph is truncated to focus on the data points that were considered in the original article from Popple et al. and the article by Desai et al. that developed the R50% guidelines.^7^

In Figure 2, one sees that many of the Falloff Index values that fell outside the proposed intermediate dose spill guidelines (UpperR50% to LowerR50%) in the UAB HyperArc data, now have R50%_FVE‐sphere_ values that fall within the proposed R50% guidelines.

## 4 DISCUSSION

Table 1 directly compares R50%_FVE_ to R50%_FVE‐sphere_. Only two columns of data (Spherical PTV SA, and R50%_FVE‐sphere_) are unique to this work, the rest of the data is directly obtained from the work of Desai and Cordrey.^6^ The Spherical PTV SA (surface area) is smaller in all cases than the Actual SA, which is expected for PTVs of irregular shapes. The final R50%_FVE‐sphere_ values compare favorably to the full R50%_FVE_ with an absolute difference of 0.82 or less for all twelve PTVs. From this, one can conclude that the values achieved by the spherical approximation FVE (R50%_FVE‐sphere_) are very comparable to the full R50%_FVE_.

The close correspondence of R50%_FVE_ and R50%_FVE‐sphere_ is due to the fact that most clinically expected targets are roughly spherical. Because larger volume objects generally have larger surface areas, the V_IDC50%_ is naturally apportioned mainly to the larger PTV in FVE. The “true R50%” for the individual PTVs in a cluster of PTVs with overlapping IDC50% cannot be known; R50%_FVE_ is an estimate. The R50%_FVE‐sphere_ is another way to estimate R50% that gives results that are comparable to full R50%_FVE_. R50%_FVE‐sphere_ is a viable option when one does not have a way to extract the PTV surface areas from the RTPS.

Turning to the clinical application of spherical PTV approximation R50%_FVE_ to the UAB dataset, we see in both Figure 2 and Table A.1 that the calculated R50%_FVE‐sphere_ is numerically less than the Falloff Index in all cases. This comes as no surprise since partitioning the overlapping IDC50% to individual PTVs by any method will give a lower value of intermediate dose spill than attributing the entire overlapping IDC50% to each individual PTV (as done in the Falloff Index). If nothing else, R50%_FVE_ allows exclusion of the volume of PTV2 when calculating R50% of PTV1 in a cluster of PTVs with overlapping IDC50%.

Not all the data in Table A.1 is visible in Figure 2 because some of the Falloff Index values reported in the UAB dataset are much larger than the displayed maximum value of 18 (the maximum value of Falloff Index in the UAB dataset is 2632). The graph was truncated at Falloff Index value 18 to focus attention on this range of moderate Falloff Index values and how these values change when re‐interpreted as R50%_FVE‐sphere_, especially relative to the proposed guidelines for R50% (UpperR50% and LowerR50%). The data is filtered to include only PTVs whose Falloff Index reported by UAB is less than 18 so that each Falloff Index point has a corresponding R50%_FVE‐sphere_ visible on the graph.

It is important to note that the difference between Falloff Index and R50%_FVE‐sphere_ can be very minimal for large PTVs, especially if a large PTV is in a cluster of PTV with much smaller PTVs. This is evident in the 35.4 mm diameter PTV (plan index = 178, PTV volume = 23.14 cm^3^), and the 27.88 mm diameter PTV (plan index = 98, PTV volume = 13.81 cm^3^). For these large PTVs the data points on Figure 2 overlap such that the blue circle of R50%_FVE‐sphere_ almost completely eclipses the red triangle of the UAB Falloff Index making the Falloff Index data point very difficult to see on the graph.

Some of the R50%_FVE‐sphere_ values were reduced by extreme amounts relative to the Falloff Index, for example, seven PTVs have a factor of 20 or greater decrease of the R50% relative to the Falloff Index; the largest decrease was a factor of 92.8. These huge reductions in numerical value are all for very small PTVs of V_PTV_ < 0.12 cm^3^, but not all PTVs with V_PTV_ < 0.12 cm^3^ experience such dramatic decrease. If there are two or more very small PTVs (V_PTV_ < 0.12 cm^3^) in an overlapping IDC50% cluster (as is seen in Plan Index = 205, Target Volumes = 0.006 cm^3^ and 0.007 cm^3^), the factor decrease is more modest, for example, a factor of 2 decrease between Falloff Index to R50%_FVE‐sphere_. In general, this very modest change from Falloff Index to R50%_FVE‐sphere_ would be anticipated any time two PTVs in a cluster with overlapping IDC50% have similar volumes. This is not necessarily a feature of small PTVs but rather a result of the similar volumes and would also be anticipated in a 2‐PTV cluster with overlapping IDC50% when the PTV volumes are large but similar.

Conversely, one anticipates large differences between R50%_FVE‐sphere_ and Falloff Index for a PTV cluster involving a large PTV in combination with much smaller PTVs. This is evident in Plan Index 178 and 17. For Plan Index 178, we see PTV volumes 0.078 and 21.1 cm^3^ where the change between Falloff Index and R50%_FVE‐sphere_ is a factor of 64 for the small PTV (0.078 cm^3^) and 1.0 for the large PTV (21.1 cm^3^). For Plan Index 17, a 4‐PTV cluster, the large PTV (volume 6.03 cm^3^) and the smallest PTV (volume 0.0147 cm^3^) show changes between Falloff Index and R50%_FVE‐sphere_ of 1.1 and 92.8, respectively. Appropriately, in these clusters with a large PTV and much smaller PTVs, R50%_FVE_ assigns most of the overlapping IDC50% to the large PTV. The large PTV sees a very small change in numerical value between Falloff Index and R50%, while the smallest volume PTV incurs the largest change from Falloff Index to R50%_FVE‐sphere_.

The clusters that contain four PTVs are more likely to manifest very large numerical differences between R50%_FVE‐sphere_ and the UAB reported Falloff Index. This is also an anticipated result because the IDC50% is partitioned between more PTVs.

## 5 CONCLUSION

This study provides a useful demonstration of the Spherical PTV Approximation of FVE for R50% (R50%_FVE‐sphere_) when the surface area of the stereotactic cranial targets is unknown. R50%_FVE‐sphere_ is applied to clinical data from the University of Alabama, Birmingham (UAB) for the 11% of cases where the 50% isodose clouds of multiple targets overlap because the PTVs are in close geometric proximity. This study re‐interprets the intermediate dose spill of this robust, useful clinical data set and provides more appropriate values for the intermediate dose spill for those 11% of cases in terms of R50% that are easier to interpret. We encourage further evaluation of the FVE method by the radiation oncology community.

## AUTHOR CONTRIBUTIONS

All authors contributed equally to this project.

## CONFLICT OF INTEREST STATEMENT

The authors report no conflicts of interest.

## Data Availability

The data that support the findings of this study are available from the corresponding author upon reasonable request.

## APPENDIX A COMPLETE DATA TABLE

Table A.1 contains full tabulation of the data for the 68 PTVs (roughly 11% of PTVs) in the UAB HyperArc dataset that have overlapping IDC50%. Except for the R50%_FVE‐sphere_ and Sphere Surface Area values calculated in this study, all the data listed come directly from the table given by Popple et al.^5^ “Plan Index” and “Target Index” are identifiers from the UAB data table.

**TABLE A1:** UAB HyperArc data for PTVs with overlapping IDC50%.

Plan Index	No. of PTVs in ISO	No. of PTVs with shared IDC50%	Target index	Target volume (cm^3^)	Target diameter (mm)	Sphere surface area (cm^2^)	Shared IDC50% Vol (cm^3^)	Falloff index UAB	R50%_FVE‐sphere_	FI UAB/ R50%_FVE‐sphere_
17	20	2	14	0.027262	3.7	0.43	1.1619	42.62	21.31	2.0
			18	0.027262	3.7	0.43		42.62	21.31	2.0
		4	5	0.095591	5.7	1.02	36.72596	384.20	20.07	19.1
			6	0.117052	6.1	1.17		313.76	18.91	16.6
			8	5.163477	21.4	14.38		7.11	6.72	1.1
			15	1.915558	15.4	7.45		19.17	8.65	2.2
		4	3	0.11266	6.0	1.13	36.66292	325.43	14.81	22.0
			13	6.031834	22.6	16.04		6.08	5.32	1.1
			16	0.014729	3.0	0.28		2489.17	26.83	92.8
			19	0.363625	8.9	2.49		100.83	10.72	9.4
24	12	2	8	0.181584	7.0	1.54	8.43988	46.48	24.50	1.9
			9	0.155278	6.7	1.41		54.35	25.70	2.1
		2	1	0.113863	6.0	1.13	2.99138	26.27	8.72	3.0
			2	0.285913	8.2	2.11		10.46	6.99	1.5
		3	10	3.191618	18.3	10.52	28.16169	8.82	5.59	1.6
			11	1.376383	13.8	5.98		20.46	6.81	3.0
			12	0.108664	5.9	1.09		259.16	13.17	19.7
78	2	2	1	7.008931	23.7	17.64	41.97495	5.99	3.52	1.7
			2	4.562679	20.6	13.32		9.20	3.80	2.4
85	10	2	6	0.184382	7.1	1.58	5.30719	28.78	25.81	1.1
			7	0.007939	2.5	0.20		668.50	69.14	9.7
		3	1	0.926772	12.1	4.60	8.02162	8.66	5.71	1.5
			2	0.353393	8.8	2.43		22.70	7.12	3.2
			3	0.024692	3.6	0.41		324.87	14.07	23.1
98	5	2	1	13.808048	29.8	27.88	44.74236	3.24	3.06	1.1
			2	0.417975	9.3	2.72		107.05	5.92	18.1
129	5	2	1	2.192471	16.1	8.14	10.76061	4.91	3.90	1.3
			2	0.41249	9.2	2.66		26.09	5.38	4.8
162	3	2	2	1.461716	14.1	6.24	15.10449	10.33	4.14	2.5
			3	2.385897	16.6	8.65		6.33	3.79	1.7
176	11	2	4	0.334969	8.6	2.32	2.49731	7.46	7.01	1.1
			11	0.008053	2.5	0.20		310.11	18.51	16.8
178	13	2	2	0.679395	10.9	3.73	8.81655	12.98	6.25	2.1
			3	0.748736	11.3	4.01		11.78	6.11	1.9
		2	6	0.078631	5.3	0.88	118.54518	1507.61	23.44	64.3
			12	23.144894	35.4	39.35		5.12	5.04	1.0
		4	4	0.449011	9.5	2.83	102.6021	228.51	30.74	7.4
			5	1.617448	14.6	6.69		63.43	20.72	3.1
			10	0.051961	4.6	0.66		1974.60	60.78	32.5
			11	3.968682	19.6	12.06		25.85	15.83	1.6
205	19	2	5	0.014125	3.0	0.28	1.14043	80.74	46.11	1.8
			9	0.009304	2.6	0.21		122.57	52.58	2.3
		2	6	0.005983	2.3	0.17	1.80441	301.59	142.39	2.1
			11	0.007061	2.4	0.18		255.55	134.90	1.9
209	8	2	2	0.034601	4.0	0.50	91.0592	2631.69	124.17	19.0
			6	2.89908	17.7	9.84		31.41	29.93	1.0
216	22	2	9	0.811609	11.6	4.23	18.31544	22.57	7.12	3.2
			13	2.234454	16.2	8.24		8.20	5.61	1.5
		2	5	0.034624	4.0	0.50	2.88945	83.45	17.71	4.7
			12	0.212466	7.4	1.72		13.60	10.71	1.3
261	4	3	1	1.320081	13.6	5.81	22.18139	16.80	4.55	3.7
			2	0.679388	10.9	3.73		32.65	5.20	6.3
			4	3.302687	18.5	10.75		6.72	3.83	1.8
275	10	2	8	0.102505	5.8	1.06	1.7262	16.84	8.90	1.9
			9	0.087941	5.5	0.95		19.63	9.25	2.1
281	13	2	5	0.582066	10.4	3.40	4.14221	7.12	5.80	1.2
			6	0.084091	5.4	0.92		49.26	9.13	5.4
		3	2	0.054245	4.7	0.69	7.78504	143.52	13.92	10.3
			7	0.84473	11.7	4.30		9.22	6.84	1.3
			9	0.217092	7.5	1.77		35.86	9.60	3.7
292	20	2	18	0.046375	4.5	0.64	1.47131	31.73	21.05	1.5
			20	0.017605	3.2	0.32		83.57	28.11	3.0
		2	4	0.211063	7.4	1.72	2.29069	10.85	8.32	1.3
			5	0.042424	4.3	0.58		54.00	12.60	4.3
321	7	2	1	2.160785	16.0	8.04	20.18496	9.34	4.41	2.1
			3	2.479853	16.8	8.86		8.14	4.30	1.9
327	16	2	6	0.012055	2.8	0.25	0.74793	62.04	29.96	2.1
			10	0.013302	2.9	0.26		56.23	29.08	1.9
